# Diagnosis and management of pituitary apoplexy: a Tunisian data

**DOI:** 10.1186/s41016-023-00331-6

**Published:** 2023-07-01

**Authors:** Faten Hadj Kacem, Oumeyma Trimeche, Imen Gargouri, Dhoha Ben Salah, Nadia Charfi, Nabila Rekik, Fatma Mnif, Mouna Mnif, Mouna Elleuch, Mohammed Abid

**Affiliations:** Hedi Chaker Hospital, 3029 Sfax, Tunisia

**Keywords:** Pituitary apoplexy, Pituitary neoplasms, Neurosurgery, Dopamine antagonists

## Abstract

**Background:**

Pituitary apoplexy (PA) is defined as the hemorrhage or the infraction of a pituitary adenoma. Aiming to determine the epidemiological, clinical, paraclinical characteristics as well as management and outcomes of PA in our population, we conducted this cross-sectional study.

**Methods:**

This cross-sectional study was conducted at the Department of Endocrinology of Hedi chaker university hospital, Sfax. Data was collected from medical charts of patients with pituitary apoplexy admitted in our department between 2000 and 2017.

**Results:**

We included 44 patients with PA. Their mean age was 50 ± 12.6 years. Among them, 31.8% had a known pituitary adenoma, and it was in all cases a macroadenoma, predominantly a prolactin secreting tumor (42.8%). A triggering factor of PA was encountered in 31.8% of cases and it was mainly: head trauma, dopamine antagonists, and hypertension. The clinical presentation of PA encompassed headaches (84.1%), visual disturbances (75%), and neurological signs (40.9%). Gonadotropin deficiency was the most frequent form of hypopituitarism noted (59.1%), followed by corticotropin deficiency (52.3%), thyrotropin deficiency (47.7%), and somatotropin deficiency (2.3%). Hormonal assessment at PA onset, concluded that 23 had a secreting adenoma: 18 prolactinomas, 3 ACTH-secreting adenomas, and 2 GH-secreting adenomas. In the 21 remaining cases, the tumor was non-functioning (47.7%).

Pituitary MRI was performed in 42 cases (95.5%), revealing infraction and or hemorrhage in the pituitary gland in 33 cases; a heterogenous signal or a fluid level within the adenoma, in nine cases.

Urgent administration of intra venous hydrocortisone was required in 19 cases. Mannitol administration was mandatory in a patient who had severe intracranial hypertension. Surgical management of the PA was imperative in 24 patients (54.5%): 15 suffered from severe visual impairment, 4 had an intracranial hypertension, 2 cases demonstrated an impaired consciousness, 2 patients experienced a tumor enlargement and one case had a severe Cushing’s disease. Operative complications found were rhinorrhea attributable to cerebral spinal fluid leakage, insipidus diabetes associated with rhinorrhea, isolated insipidus diabetes, and hydrocephalus in one case each. Long-term follow-up concluded that headaches persisted in five cases, owing to the tenacity of a macroprolactinoma regardless of cabergoline treatment in one case, the recurrence of an adenoma in two cases and its persistence despite the medical and the surgical treatment in two patients. Concerning the visual acuity defects, only two patients had persistent diminished visual acuity at long-term follow-up.

Among 25 patients, 13 were diagnosed with definitive thyrotropin deficiency. Similarly, 14 patients had persistent corticotropin deficiency (CD). Additionally, CD was de novo diagnosed in two patients. Otherwise, gonadotropin deficiency prevailed in all cases. Persistent prolactin deficiency was seen in two patients. Disappearance of the pituitary tumor was encountered in 11 out of 24 cases at long-term follow-up. Overall, surgery was associated with better outcome than conservative management.

Pituitary apoplexy is a challenging condition due to its variable course, its diagnosis difficulty and management, as gaps remain to determine the best approach to treat this condition.

**Conclusions:**

To conclude, pituitary apoplexy is a challenging condition due to its variable course, its diagnosis difficulty and management, as gaps remain to determine the best approach to treat this condition. Further studies are thus needed.

## Background

Pituitary apoplexy (PA) is defined as the hemorrhage or the nfraction of pituitary adenoma. It is a rare disease, occurring in up to 25% of patients with pituitary adenoma [1]. The clinical manifestations of this condition ranges from asymptomatic presentation to a dramatic deterioration of consciousness, decreased visual acuity and hormonal dysfunction; depending mainly on the extent of oedema, hemorrhage and necrosis of the pituitary gland. While some scholars adopt a conservative approach others opt for a decompression surgery [2]. Given the paucity of data in our country about this pathology, we conducted this study. Our aim is to determine the epidemiological, clinical, paraclinical characteristics as well as management and outcomes of PA in our population.

## Methods

### Study design and setting

This cross-sectional study was conducted at the Department of Endocrinology of Hedi chaker university hospital, Sfax, which is the second largest city of Tunisia. Data was collected from medical charts of patients with pituitary apoplexy admitted in our department between 2000 and 2017.

### Participants

We included patients who were referred to our department with acute clinical presentation of PA, including those with headaches and/or visual disturbances and/or endocrine dysregulation. Asymptomatic patients with radiological PA were also eligible for our study. We excluded the cases of PA secondary to suprasellar lesions.

### Variables

A pre-established information sheet was made to collect epidemiological information as well as personal and familial health history of each patient.

Clinical evaluation encompassed reasons of consultation and clinical presentation.

### Hormonal assessment

All our patient underwent endocrine evaluation to identify hypopituitarism. Hormonal hypersecretion assessment was based mainly on clinical assessment. However, IGF1 (Insulin-like growth factor (1) as well as prolactin analysis was indicated in all of our patients.

Static and dynamic hormonal testing were realized at the Department of Biochemistry at Habib Bourguiba hospital in Sfax. Hormonal evaluation included the evaluation of the thyroid axis (thyrotropin stimulation hormone: TSH and free thyroxine: FT4), the gonadotropic axis (testosterone for men, estrogen for women and Follicle stimulating hormone: FSH, luteinizing hormone: LH, in both sex), the corticotropic axis (morning cortisol and adrenocorticotropic hormone: ACTH), the somatotropic axis (growth hormone: GH and IGF1) and prolactin level. Dynamic tests were only used in some specific cases, either to identify hormonal deficiency or hypersecretion.

#### Corticotropic axis evaluation

Corticotropin deficiency (CD) corresponds to a low 8 am cortisol (< 50 ng/ml) associated with inappropriate level of ACTH (increased or normal). This latter diagnosis was ruled out when 8 am cortisol was over 180 ng/ml. For intermediate cortisol levels ranging between 50 and 180 ng/ml, a 1-µg ACTH stimulation test was performed. Peak cortisol below 180 ng/ml 30 or 60 min after ACTH stimulation test confirmed the diagnosis of CD. The gold standard test for the diagnosis of CD is the insulin tolerance test which consists of the intravenous injection of insulin 0.1 ui/kg. A peak cortisol level above 180 ng/ml obtained after hypoglycemia excluded CD.

A Cushing’s syndrome is suspected when 8 am cortisol level is above 18 ng/ml after low-dose overnight dexamethasone, whereas it is confirmed once 8 am cortisol remains above 18 ng/ml after high-dose overnight dexamethasone. A central origin of the hypercortisolism is based on an ACTH level over 15 pg/ml.

#### Somatotropic axis evaluation

Low IGF1 level made the diagnosis of growth hormone deficiency. A GH nadir after OGTT superior to 1 ng/ml established the diagnosis of Acromegaly.

#### Gonadotropic axis evaluation

As for gonadotropin deficiency, it is defined as inadequate levels of gonadotrophins (FSH and LH) associated with low estradiol and low testosterone in women and in men, respectively.

#### Thyrotropic axis evaluation

Thyrotropin deficiency corresponds to a low FT4 associated with an inappropriate TSH level (decreased or normal).

#### Lactotroph axis evaluation

The diagnosis of hyperprolactinemia consists of two prolactin measurements above 25 ng/ml, after excluding other diagnosis such as medications.

### Ophthalmological assessment

The vast majority of our patients had visual field and/or dilated fundus examination, before treatment and during follow-up if they remained symptomatic.

### Radiological assessment

All patients had neuroimaging either CT scan and/or MRI. The diagnosis of PA is based on the presence of infraction and or hemorrhage on one or two of these latter imaging modalities.

### Management and outcomes

Detailed medical and or surgical management of the PA was specified for each patient.

The standard approach for patients with severe neuroophthalmological complications of PA is urgent decompressive surgery. Otherwise, it is an initial watchful monitoring. Differed surgery is indicated for patients with ophthalmological and or neurological compromise or hypersecreting pituitary tumors besides prolactinomas.

Positive outcome of treatment is defined as an improvement of the initial symptomatology and or the fading of the adenoma and or the resolution of hypopituitarism.

As for follow-up, pituitary MRI, visual field testing and hormonal assessment for hypopituitarism is recommended for most of our patients at 3 months, 6 months after initial management and yearly afterwards.

### Statistical methods

Statistical analysis was performed using the 20th version of SPSS. Categorical variables are detailed as frequency or percentage and were compared via chi-square or Fisher’s exact test, as appropriate. Continuous variables as median with interquartile range, or mean with standard deviation and were analyzed using paired/unpaired *t* test, analysis of variance (Bonferroni post hoc test), Mann–Whitney test, Kruskal–Wallis test (Dunn’s post hoc test), and Wilcoxon, as suitable.

## Results

### Participants

A total of 48 cases of PA were found in the register of admissions in our department from 2000 to 2017. Among them, four were excluded as they were illegible to our study.

### Outcome data

#### Epidemiological characteristics

The mean age of our patients was 50 ± 1.6 years, with extremities ranging from 26 to 75 years. Sex ratio was 1,2 (Table 1). Before the age of 50 years, this ratio was one. Most of our study population lived in urban areas (58.5%).

**Table 1:** Epidemiological and clinical characteristics of our study population

	**Number of patients**	**Frequency** (%)
Men	24	54.5%
Smoking	15	34.1%
Alcohol consumption	5	11.3%
Personal history of pituitary tumor	14	31.8%
Headaches	37	84.1%
Decreased visual acuity	16	36%
Visual field defects	16	36%
Diplopia	11	25%
Neurological signs	18	40.9%
Signs of hormonal hypersecretion	13	29.5%

#### Clinical characteristics

Among our patients, only one had a family history of pituitary disease.

As for personal history: 11 had type 2 diabetes (25%), 7 were hypertensive (15.9%), and 4 had dyslipidemia (9.1%). Additionally, most of our patients were smokers (34.1%), and five consumed on a regular basis alcohol (11.3%) (Table 1). Otherwise, fourteen had a known pituitary adenoma before having a PA (31.8%). It was in all these cases a macroadenoma: a prolactin secreting tumor in six cases, two corticotroph adenoma, one somatotroph adenoma and non-functioning adenoma (NFA) in the remaining cases. Surgical treatment was required in a case of corticotroph adenoma, which relapsed 5 years later. A patient with a non-functioning adenoma was operated three times but the tumor recurred 9 years later. Two patients refused the surgical procedure. Conservative management was conducted in 10 cases: dopaminergic agonists in seven patients and substitutive treatment with l-thyroxine and oral hydrocortisone in three.

PA was revealing of the adenoma in 30 cases (68.2%). Else, it was discovered incidentally in two cases on brain imaging performed for other reasons: maxillary lesion and malignant staphylococcal infection of the face.

A triggering factor of PA was found in 14 cases (31.8%). Table 2 summarizes the main precipitating factors incriminated in the reveal of PA.

**Table 2:** Main triggering factors of pituitary apoplexy

**Triggering factor**	**Number of patients** (***n*** = 14)	**Frequency** (%)
Head trauma	4	28.6
Cabergoline	3	21.4
Bromocriptine	1	7.1
Hypertensive emergency	2	14.3
Major surgery	1	7.1
Post-partum	1	7.1
Anticoagulant	1	7.1
Infection	1	7.1

Headaches were the most frequent symptomatology found in our patients (84.1%). These headaches were: of sudden-onset (57.1%), intense (64.3%), holocranial (50%) and paroxystic (85%). Visual disturbances were observed in 33 cases (75%) and consisted of: decreased visual acuity (36%), visual field defects (36%), diplopia (25%), blurred vision (9%) and cecity (6.8%). As for neurological signs, they were present in 18 cases (40.9%) (Table 1). A franc intracranial hypertension syndrome was observed in 15 cases (31.1%), consisting of rebellious headaches, vomiting and visual defects. It was associated with a meningeal syndrome in four cases, agitation and obnubilation in one case each. Otherwise, an isolated altered mental status was noted in two cases, and one patient had obnubilation along with right hemiparesis.

Amongst our patients, 13 exhibited signs of hormonal hypersecretion: an amenorrhea-galactorrhea syndrome (four cases), a gynecomastia (two cases), hypercortisolism signs (three cases) and acromegaly features (four cases) (Table 1).

Else, 34 of our patients had an ophthalmological examination (77.3%). A decreased VA was noted in 10 patients (22.7%) with varying degrees of severity ranging from slightly diminished VA to complete monophtalmic cecity. Bitemporal hemianopsia was observed in 13 patients (29.5%) and a visual field defect was detected in four cases (11,7%). Papillary oedema, it was found in five cases (14.7%). Optic atrophy was seen in one case. Third nerve palsy was noticed in seven cases (15.9%). As for fourth and sixth nerve palsy, they were noted in four and two cases, respectively.

At PA onset, gonadotropin, thyrotropin and corticotropin deficiencies were the most frequently encountered hormonal dysfunction: 59.1%, 56.8%, and 52.3%, respectively. Concerning prolactin and somatotropin deficiencies, they were detected in four (9.1%) and in two cases (4.5%), respectively.

Hormonal assessment at PA outbreak, concluded that 23 had a secreting adenoma: 18 prolactinomas, three ACTH-secreting adenomas and two GH-secreting adenomas. The tumor was non-excreting in 21 patients (47.7%).

**Fig. 1:**
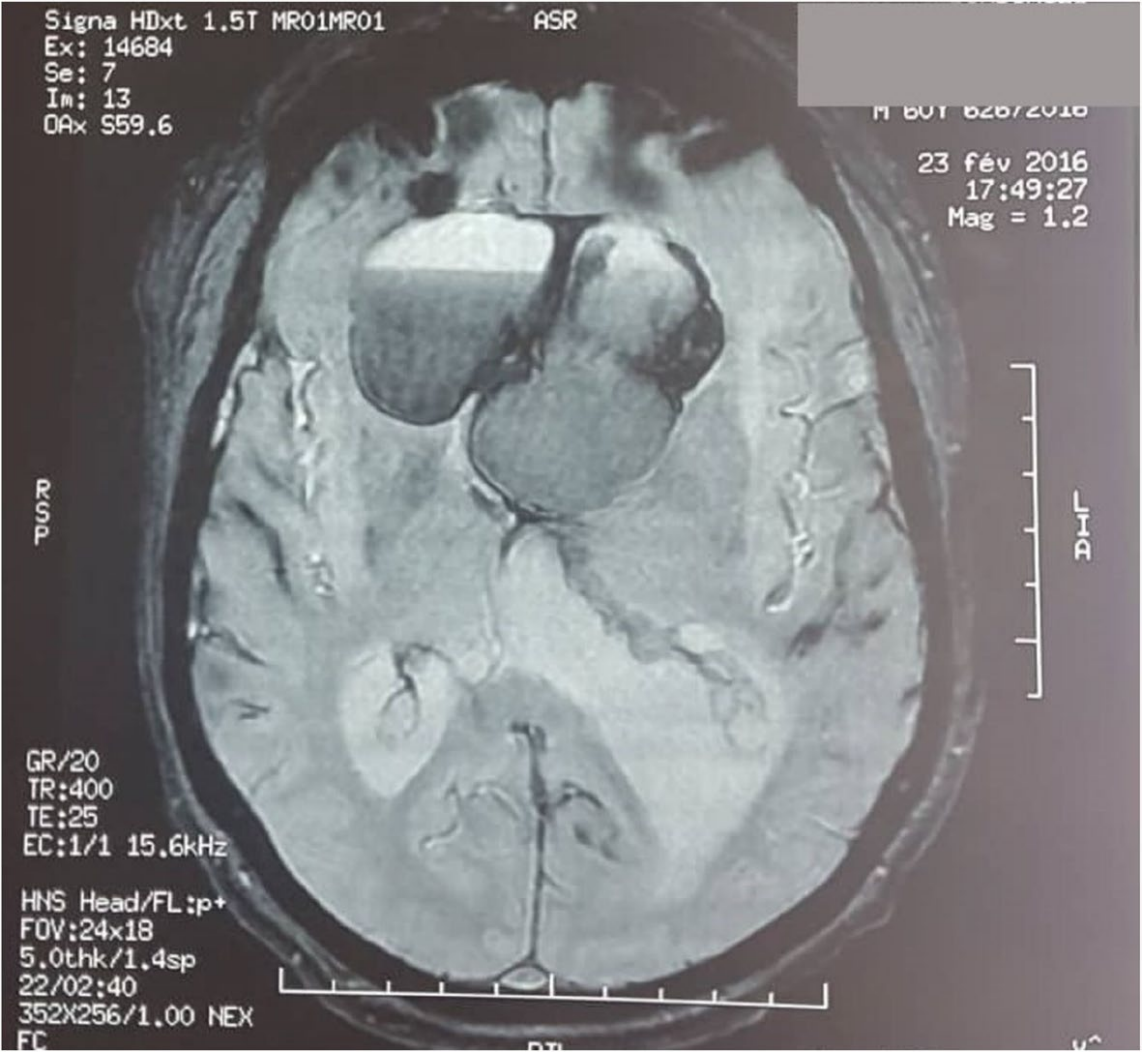
The fluid level of an adenoma

Cerebral CT was performed in 24 cases (54.5%). It revealed an enlarged sella turcica and osteolysis in four cases, each. A pituitary MRI was carried out in 42 cases (95,5%), revealing a necrosis and or a hemorrhage in the pituitary gland in 33 cases and an heterogenous signal or a fluid level within the adenoma (Fig. 1), in 9 cases.

Urgent administration of iv hydrocortisone was required in 19 cases. One patient with severe intracranial hypertension warranted Mannitol administration. Hormonal replacement therapy with hydrocortisone and levothyroxine was needed in 23 and 21 cases, respectively. Dopaminergic agonists were indispensable for the 18 patients with prolactinoma: 10 patients were treated with Bromocriptine whereas 8 were prescribed with Cabergoline.

Surgical management of PA was imperative in 24 patients (54.5%): 15 had a severe visual impairment, 4 an intracranial hypertension, an impaired consciousness in 2 cases, a tumor enlargement in 2 cases and 1 case of severe Cushing’s disease. Table 3 resume the visual impairment indicating surgery in our study.

**Table 3:** Visual defects indicating surgery in our study

Visual defect	Number of patients
Papillary oedema	5
Monocular cecity	1
Ophthalmoplegia	2
Visual field defect with decreased visual acuity	8

Surgery consisted in all cases of a transsphenoidal approach. The median delay between surgery and the apoplectic episode was 74.5 ± 47.6 days. In seven cases, surgery was performed in the week following the onset of the PA. Operative complications observed were rhinorrhea attributable to cerebral spinal fluid leakage, insipidus diabetes associated with rhinorrhea, isolated insipidus diabetes, and hydrocephalus in one case each. Despite the external ventricular bypass, the patient with hydrocephalus deceased.

Results of the immunochemistry examination of the pituitary tumor are detailed in Table 4.

**Table 4:** Results of immunochemistry examination on the resected tumors

Nature	Number of patients	Percentage
PRL	17	38.6%
GH	5	11.4%
ACTH	3	6.8%
PRL + GH + ACTH	4	9.1%
PRL + GH	2	4.55%
GH + ACTH	2	4.55%
Non-functioning	11	25

The mean follow-up duration was 43.7 months (0.06–204). One patient with severe auriculo ventricular block deceased before the surgery.

Long-term follow-up concluded that headaches persisted in five cases, attributable to a macroprolactinoma with apoplexy resistant to cabergoline in one case, recurrence of the adenoma in two cases, persistence of the adenoma despite appropriate medical and surgical treatment in two cases. Concerning the visual acuity defects, only two patients had persistent diminished visual acuity at long-term follow-up. A diplopia was found in one patient who had an empty Sella turcica.

Among 25 patients, 13 were diagnosed with definitive thyrotropin deficiency. Similarly, a persistent CD was noted in 14 cases. Additionally, a CD was de novo diagnosed in two patients. Otherwise, gonadotropin deficiency prevailed in all cases. Only two patients had persistent prolactin deficiency. Twenty-four patients had an MRI at follow-up. The results are displayed in Table 5.

**Table 5:** MRI data at follow-up

MRI delay after the onset of the PA	Number of patients	Persistence or recurrence of the pituitary adenoma		Disappearance of the pituitary adenoma	
1 month	4	3	4.5%	1	4.5%
3 months	8	5	11.4%	3	6.8%
6 months	7	6	13.6%	1	2.3%
1 year	14	9	20.5%	5	11.4%
Long term	10	5	11.4%	5	11.4%
Last MRI	24	13	31.8%	11	20.5%

When comparing management outcomes which encompasses resolution of hypopituitarism, visual disturbances and neurological signs between patients who had an urgent surgery (before 7 days), deferred surgery and conservative approach, we found that significant results were only perceived for visual impairment. In fact, post hoc analysis revealed that the resolution of visual impairment was more frequent among those who had urgent surgery compared to the two other approaches (*p* = 0.024).

Overall, positive outcome was mainly observed after surgical treatment as 9 out of 22 patients (40.9%) after a mean follow-up of 4.4 years, versus 2 out of 15 cases (13.3%) who had a conservative treatment.

## Discussion

To date, the management of PA is still a matter of debate. To address this issue and to determine the clinical and paraclinical characteristics of PA in our population, we conducted this study. Our work generated interesting results.

The mean age of our study group was 50 years. It was slightly more encountered in men (55%). These findings are consisting with the literature, as PA are mostly seen between the 5th and the 6th decade and is more frequent in men [1].

One third of our patients had a known pituitary adenoma before the onset of the PA, and it was in all these cases a macroadenoma. A triggering factor was identified in 31.8% of cases and the leading predisposing factors to PA found were head trauma, cabergoline use, and hypertension. Similar results were observed in other studies, as the precipitating factors were determined in 10 to 40% of cases of PA [1, 3]. The pathophysiology of PA is yet to be ascertained. However, some authors suggest that the low perfusion rate of portal vessels which irrigate the adenoma can explain in part their susceptibility to infraction [4]. Additionally, some research suggest that the adenoma outgrows their blood perfusion which results in its infraction [5].

In the setting of PA, headaches were the revealing symptom in most cases (84.1%). Visual defects were noted in 75% of the cases and consisted of decreased visual acuity and visual field defects in most cases. As for neurological signs, they consisted mainly of an intracranial hypertension syndrome. Hypopituitarism was observed in more than half of cases and it involved mostly gonadotropin, thyrotropin, and corticotropin deficiency. Conversely, other studies demonstrated a more prevalent GH deficiency [1]. The discrepancy of results between our study and other reports can be explained by the fact that GH testing was done in only few patients and it was searched for using IGF1, which lacks sensitivity for the detection of GH deficiency.

In the majority of cases, the adenoma was non-functioning (47.1%) which is in line with other studies [6–8]. This finding may be attributable to the fact that NFA are asymptomatic, diagnosed late and hence their voluminosity and susceptibility to apoplexy. Additionally, Cardoso et al. suggest that a vasculopathy of pituitary adenoma contributes to their proneness to hemorrhage and infraction. This theory is endorsed by the over expression of vascular endothelial growth factor (*VEGF*) in tissues emanated from NFA [9]. Thereby and besides the latter hemodynamic and mechanical hypothesis cited above, an intrinsic structural vasculopathy could be added as a possible mechanism of PA.

Otherwise, ACTH-secreting adenoma are found to be more prone to apoplexy as it was established by many reports: in fact, it occurs in 30 to 60% of cases compared with 2 to 14% of other types of adenomas [1, 10–12]. This may be related to the effect of acute release of ACTH, which causes hypertension, hyperglycemia, and edema, and therefore apoplexy [1].

CT scan is generally the first used brain imaging in the setting of PA, due to its availability. It enables the diagnosis of pituitary mass and depicts recent hemorrhage [1]. MRI, however, is the modality of choice in diagnosing PA, as it detects hemorrhage in its different stages as well as ischemia [13]. The MRI signal varies with the different stages of degradation of hemoglobin which helps in estimating the outbreak of hemorrhage.

The aftermath of PA is variable as it depends mainly on the histological components of the adenoma. Infraction is usually associated with a better prognosis and possible spontaneous involution of the tumor whereas hemorrhagic infraction or hemorrhage leads to a more severe clinical presentation and a worse outcome [1]. Given the variability of outcome, management of PA is still controversial, albeit the reestablishment of electrolyte balance and hormonal replacement, which is consensual. Owing to the relative frequency and the life-threating nature of CD, it is actually recommended to treat patients with PA with iv corticosteroids [2].

Pituitary surgery was mandatory in 54.4% of cases in our study considering the pronounced degradation of visual function in most cases, the intracranial hypertension, the altered consciousness, the tumor enlargement and the severe Cushing’s disease. Similarly, current guidelines suggest performing decompression surgery in patients with serious neuro-ophthalmological symptoms [2]. Conservative management based on steroids is another option for the management of PA, indicated in the absence of severe neurological and ophthalmological compromise. Nevertheless, controversy on the management of PA resides in the exact definition of these latter symptoms [2].

Overall, endocrine function is persistently altered after PA even when surgery was performed [13]. However, some authors suggest that early surgical intervention could enhance endocrine outcome, as it is the consequence of compression effect of the adenoma on the pituitary gland and not the gland necrosis and thus an early decompression surgery is capable of improving endocrine function in these patients [14, 15]. Otherwise, surgery is a rapid solution to resolve ophthalmologic symptomatology of PA as it enables the improvement of the visual function in up to 85% of patients and its total recovery in 38% of cases [16]. Timing in these cases is a major issue to consider, if indicated, surgery must be performed in the week following the subset of the PA. Headaches are also substantially approved after surgery. Despite the possible positive outcomes of this latter treatment modality, it may be complicated with cerebral spinal fluid leakage and thus meningitis, insipidus diabetes and hypopituitarism [1]. Therefore, Pros and cons of each approach should be discussed with a multidisciplinary team.

## Conclusion

To conclude, pituitary apoplexy is a challenging condition due to its variable course, its diagnosis difficulty and management, as gaps remain to determine the best approach to treat this condition. Further studies are thus needed.

## Data Availability

All data generated or analyzed during this study are included in this published article.

## Abbreviations

PA: Pituitary apoplexy
TSH: Thyrotropin stimulation hormone
FT4: Free thyroxine
FSH: Follicle stimulating hormone
LH: Luteinizing hormone
ACTH: Adrenocorticotropic hormone
GH: Growth hormone
IGF1: Insulin-like growth factor 1
CD: Corticotrope deficiency

## References

[1] Briet C, Salenave S, Bonneville JF, Laws ER, Chanson P (2015). Pituitary Apoplexy. Endocr Rev..

[2] Rajasekaran S, Vanderpump M, Baldeweg S, Drake W, Reddy N, Lanyon M (2011). UK guidelines for the management of pituitary apoplexy. Clin Endocrinol (Oxf)..

[3] Ranabir S, Baruah MP (2011). Pituitary apoplexy. Indian J Endocrinol Metab sept.

[4] Bjerre P, Gyldensted C, Riishede J, Lindholm J (1982). The empty sella and pituitary adenomas. A theory on the causal relationship.. Acta Neurol Scand..

[5] Semple PL, Webb MK, de Villiers JC, Laws ER (2005). Pituitary apoplexy. Neurosurgery..

[6] da Motta LA, de Mello PA, de Lacerda CM, Neto AP, da Motta LD, Filho MF (1999). Pituitary apoplexy. Clinical course, endocrine evaluations and treatment analysis. J Neurosurg Sci..

[7] Falhammar H, Tornvall S, Höybye C. Pituitary Apoplexy: A Retrospective Study of 33 Cases From a Single Center. Front Endocrinol. 2021;12:656950. 10.3389/fendo.2021.656950 PMC8082680 33935971

[8] Bills DC, Meyer FB, Laws ER, Davis DH, Ebersold MJ, Scheithauer BW (1993). A retrospective analysis of pituitary apoplexy. Neurosurgery.

[9] Cardoso ER, Peterson EW (1984). Pituitary apoplexy: a review. Neurosurgery.

[10] Scheithauer BW, Jaap AJ, Horvath E, Kovacs K, Lloyd RV, Meyer FB (2000). Clinically silent corticotroph tumors of the pituitary gland. Neurosurgery.

[11] Webb KM, Laurent JJ, Okonkwo DO, Lopes MB, Vance ML, Laws ERJ. Clinical Characteristics of Silent Corticotrophic Adenomas and Creation of an Internet-accessible Database to Facilitate Their Multi-institutional Study. Neurosurgery. 2003;53(5):1076. 10.1227/01.neu.0000088660.16904.f7 14580274

[12] Choudhry OJ, Choudhry AJ, Nunez EA, Eloy JA, Couldwell WT, Ciric IS (2012). Pituitary tumor apoplexy in patients with Cushing’s disease: endocrinologic and visual outcomes after transsphenoidal surgery. Pituitary.

[13] Muthukumar N (2020). Pituitary apoplexy: a comprehensive review. Neurol India..

[14] Randeva HS, Schoebel J, Byrne J, Esiri M, Adams CB, Wass JA (1999). Classical pituitary apoplexy: clinical features, management and outcome. Clin Endocrinol (Oxf)..

[15] Hosmann A, Micko A, Frischer JM, Roetzer T, Vila G, Wolfsberger S (2019). Multiple pituitary apoplexy-cavernous sinus invasion as major risk factor for recurrent hemorrhage. World Neurosurg.

[16] Zoli M, Milanese L, Faustini-Fustini M, Guaraldi F, Asioli S, Zenesini C (2017). Endoscopic endonasal surgery for pituitary apoplexy: evidence on a 75-case series from a tertiary care center. World Neurosurg.

